# “Out of Touch”—Recovering Sensibility after Burn Injury: A Review of the Literature

**DOI:** 10.3390/ebj3020032

**Published:** 2022-06-14

**Authors:** Savas Tsolakidis, Ziyad Alharbi, Hans Oliver Rennekampff, Markus Robert Schmidhammer, Robert Schmidhammer, Rudolf Rosenauer

**Affiliations:** 1 Austrian Cluster of Tissue Regeneration, Research Centre for Traumatology of the Austrian Workers Compensation Board (AUVA), Ludwig Boltzmann Institute for Experimental and Clinical Traumatology, Donaueschingenstraße 13, 1200 Vienna, Austria; r.schmidhammer@icloud.com (R.S.); rudolf.rosenauer@auva.at (R.R.); 2 Millesi Center for Surgery of Peripheral Nerves, Vienna Private Clinic, Pelikangasse 15, 1090 Vienna, Austria; 3 Plastic Surgery and Burn Unit, Dr. Soliman Fakeeh Hospital, Jeddah 23323, Saudi Arabia; zialharbi@fakeeh.care; 4 Klinik für Plastische Chirurgie, Hand-und Verbrennungschirurgie, Rhein Maas Klinikum, Mauerfeldchen 25, 52146 Wuerselen, Germany; orennekampff@gmail.com; 5 Klinik für Neurochirurgie, Universitaetsklinikum Ulm, Albert-Einstein-Allee 21, 89081 Ulm, Germany; markus.schmidhammer@uniklinik-ulm.de; 6 Trauma Hospital Lorenz Boehler of the Austrian Workers’ Compensation Board (AUVA), Donaueschingenstrasse 13, 1200 Vienna, Austria

**Keywords:** burn injury, full-thickness burn injury, sensory regeneration, cutaneous sensibility, nerve injury, nerve regeneration, skin grafting

## Abstract

Background: Full-thickness burn injuries (FTBI) not only lead to a significant burden in multiple ways, including social life and self-esteem, but have also a tremendous impact on environmental interaction by reducing sensibility in manifold ways. On these grounds, possible ways and solutions to recover sensibility in burn wounds are essentials and should not be overlooked. Methods: A review of experimental, clinical studies and the related literature was performed with the aim to highlight post-burn nerve regeneration and discover ways for sensory re-integration to complement the therapeutic concept. Results: In human burn injuries, it has been hypothesized that grafted cells, partly multipotent stem cells, could be additionally responsible for nerve regeneration in burn wound areas. In addition, burn eschar excision, performed within a short post-burn time frame, can reduce or even avoid long-term nerve damage by reducing post-burn toxic mediator release. Various animal studies could demonstrate sensory reinnervation of different qualities in burn wounds. Post-burn scar tissue prevents, or at least decelerates, nerve reinnervation, but could be reduced by targeted mediators. Conclusion: Sensory loss is present in skin grafted areas following full-thickness burn-wound excision, thereby leading to a reduction in quality of life. In addition, various mediators might reduce or avoid nerve damage and should be considered at an early stage as part of a holistic burn-patient therapeutic approach. In addition, supportive multifaceted physical therapy strategies are essential.

## 1. Introduction

Losing skin’s natural barrier, as a result of a burn injury, can lead to deleterious effects to the whole body system as less than 15% of the total body surface area (TBSA) can be life threatening by initiating massive water loss, hypermetabolism, and immunodepression.

Depending on the depth of the burn injury, all or parts of the skin, including sensory receptors and the appendages, will be destroyed. The full extent of tissue and physical damage may not always be evident immediately after a burn injury [1,2,3,4].

Superficial partial-thickness burns affect the epidermis and parts of the superficial dermis, thereby, with the potential for scarless healing. Deep partial-thickness burn injuries are characterized by loss of the epidermis and deep dermal layer. Uncontrolled healing will lead to various extents of scarring. Full-thickness burns include complete damage of the skin down to the hypodermis layer and are, therefore, routinely excised and skin grafted. 

A variety of specialized nerve endings, initially appearing in the fourth fetal month [5], are located in the skin, including sensory nerve endings, responsible not only for touch, but also for perception of temperature, pain, and vibration. Meissner corpuscles, Merkel cells, Pacinian corpuscles, and free nerve endings represent the dominant mechanoreceptors and reveal an irregular pattern of distribution. Meissner corpuscles, responsible for touch, can be found between the dermal papillae. Merkel cells for the perception of pressure are located on the junctional level of the epidermis and superficial dermis and can be primarily found in the skin of the palm of the hands and soles of the feet. Pacinian corpuscles, liable for the registration of vibration, lay in the deep dermal layer. Free nerve endings can be found in all dermal layers [5,6,7,8], while one axon transfers one information afferent to the dorsal root ganglia and further to the brain.

Although cell bodies on the spinal root level are preserved after localized burn injuries, perceptions are reduced or lost in the burn site due to local destruction of receptor-assigned afferent axons. Interestingly, a loss of perception has also been reported in unburned areas [9,10]. 

“Losing Touch”, describing the severe loss of sensory perceptions, can not only lead to a significant loss of environmental interaction, but also to severe restraints of individual stress, resulting in quality-of-life reduction and biopsychosocial derangement [11]. 

Clinical tests for the sensory evaluation of the perception of various qualities consist of the von Frey hair filament test, vibrometer, tuning fork, pointy metal pen, blunt, and sensory-evoked potential test [12].

In full-thickness burn injuries (FTBI), loss of all sensitivity represents a significant burden to the burn patients. In approximately 70% of the patient population, reinnervation is imperfect, resulting in misperception and sometimes neuropathic chronic pain [9]. Post-burn allodynia, neuropathy, and neuropathic pain, first described in 1971 [13], additionally affect many burned patients, ranging from 11–52% [14,15]. Neuropathic pain, as a consequence of a burn injury, is characterized as a misperception of sensibility and seems to correlate with the severity of the burn injury and length of the hospital stay itself [16]. Allodynia represents a pain characteristic, which results from a stimulus, that normally does not result in pain by itself. In many cases, dysfunctional nerve areas are ignored or neglected as part of a general and complete burn-injury treatment approach [15,16].

The sequelae of sensory derangement are not well understood. In general, the regeneration of nerves derives from uninjured deep tissue layers as well as the wound-bed area [17]. For superficial partial-thickness burn injuries, Malenfant et al. reported that sensibility can completely recover, except for touch/pressure and perception of heat [10]. Nerve reinnervation and, thus, the return of sensibility and perception in the burn area also depend on the choice of treatment; skin grafting, which represents the gold standard in terms of treating FTBI, may result in higher rates of paresthesias and malsensation [10].

Other authors stated that skin grafting leads to nerve regeneration from the deep tissue layers, as well as collateral sprouting from intact nerve fibers from the wound edge and grafted cells. However, healing by secondary intention with subsequent scar formation impairs nerve regeneration in post-burn skin [18].

The question remains how clinicians can improve nerve-fiber reinnervation, especially in cutaneous burn injuries where skin grafting is necessary. The purpose of this narrative review is to focus on post-burn sensibility impairment and identify possible experimental and clinical strategies to improve nerve reinnervation in burned areas.

## 2. Methods

We performed a literature review of studies, reporting various burn reconstruction methods and post-treatment controls.

The PubMed database was searched, irrespective of the year of publication, utilizing the following independent search terms: burn injury and nerve regeneration, burns and nerve regeneration, nerve regeneration, burns and sensibility, burns and sensory function, burn injuries and cutaneous sensibility, tissue regeneration, peripheral neuropathy and burn injuries, and peripheral neuropathy and burn injuries.

No restrictions on time nor language were implemented. Additional articles have been added to the review process after reviewing references. All articles were manually edited. The articles included were of the following type: case series, retrospective analyses, prospective analyses, double-blind studies, burn-injury animal studies, anatomical studies, and review articles. 

With such heterogenicity of publications and only one clinical report on an intervention, we did not aim to perform a systematic review or metanalysis according to PRISMA criteria.

## 3. Results

A total of 25 scientific papers, meeting the inclusion criteria regarding burns, were retrieved and analyzed. Publication dates ranged from 1925 to 2019. A total of 19 publications were added after reviewing references of the initially included papers (cross-referencing). We identified 2 human case series and 12 retrospective analyses, 2 prospective studies, 1 comment, no randomized control trials, 17 animal studies, 2 review articles, and 8 topic-related miscellaneous articles. In total, 44 publications were analyzed. Overall, a total of 3132 patients were included in the clinical human studies, ranging from 3 to 70 years old. Table S1 (Supplementary Materials) represents an Overview of the Human and Animal Studies included in the Manuscript in Alphabetical Order.

### 3.1. Animal Experiments

Experimental animal studies demonstrated a clear association between cutaneous burn injuries and reduced nociceptive function at the wound site and surrounding uninjured skin [19]. Splitting perception into single sensory qualities, the animal studies revealed that 8 weeks after full-thickness burn injury, nociceptive function is renewed, mainly in distant sites and reduced in the wound center and at the edges [20]. Saffari et al. [21] reported that 24 h after thermal insult, nerve fibers were completely absent in the wound. In their rat model, PGP 9.5- and CGRP-immunoreactive nerve fibers reappeared four weeks after the burn injury, while H- and GAP-43-IR nerve fibers remained absent. Peptidergic and non/peptidergic nerve fibers, except sympathetic noradrenergic nerve fibers, could be verified in the developed scar tissue 2 weeks after a full-thickness injury and returned to normal 8–12 weeks after burn trauma in the center of the lesion [21].

In a couple of experiments, Waris [22] analyzed the reinnervation of scars. Nerves with nonspecific cholinesterase activity were found 4 weeks after wound healing, and free nerve endings could be observed 20 weeks post-injury. Guidance of adrenergic nerve fibers by regenerating sensory nerve fibers was histologically confirmed after burn injury [22].

Kim et al. analyzed the systemic effect of cutaneous burn injury, demonstrating a denervation-like response in distant non-burned tissue, e.g., muscle [23]. The authors concluded that the observed dysfunction may account for muscle weakness. Further studies demonstrated the causal importance of elevated adenosine 3′:5′-cyclic monophosphate (cyclic AMP) levels for muscle dysfunction [19].

Higashimori et al. investigated the therapeutic effect of burn-wound excision on sensory reinnervation. In a mouse model, early excision of full-thickness burns impeded motor and sensory impairment. In contrast, delayed excision led to a significant reduction of nerve conduction, probably due to elevated serum levels of nitric oxide and TNF alpha [24]. Equally interesting, it could be demonstrated in another animal study that scar tissue development could be reduced, or even avoided, and nerve regrowth increased by IL-10 application [20].

The use of a reconstructed human skin seeded with Schwann cells, derived from multipotent mesenchymal stem cells, showed promising results. Histology revealed the formation of Büngner band-like structures. The study undermined the possibility to increase nerve reinnervation by a tissue-engineering approach [25].

### 3.2. Human Studies

Histopathological studies demonstrated that skin-grafted sites and post-burn scars depict a similar reinnervation pattern with immuno-reactive nerve fibers, along with complex sensory receptors in the dermal papillae and deep dermis [26]. Sensibility correlated with the number of nerve structures. In a retrospective analysis of 121 patients with healed burns, a significant sensory loss, not only in the burn sites but also in the non-injured areas, more than 18 months post-burn have been revealed [10]. A similar result of elevated threshold in the unburned skin of burned patients was described by Ward and Tuckett [9]. The authors also reported that functional deficits correlated with burn depth. Nedelec et al. also pointed out that that innervation in skin-grafted sites does not return to normal values. Even in burn scars after spontaneous healing, reduced innervation was found [27]. In contrast to these studies, Pontén in 1960 reported that burn patients with grafted skin could differentiate regular perception of tactile, thermal, and pain one year after burn injury [18]. In a recent retrospective analysis of 31 children with FTBI and skin grafting, over 90% regained both touch and vibration [12]. In children with FTBI and subsequent skin grafting (split thickness vs. full thickness), almost all regained sensibility in terms of different qualities, meaning mechano-reception and vibration reception. Hermanson described in 1986 that even in superficial burn injuries, sensory regaining was incomplete, especially regarding thermal sensibility [28]. Another retrospective human study analyzed burned adult patients and revealed a significant reduction of touch, temperature, and a numerical reduction of blood vessels and sweat glands, even 3 years after their injury [27].

A variety of predictive factors for developing post-burn neuropathy has been reported. These include age > 20 years, TBSA > 20%, and intensive care unit stay > 20 days [15,29,30]. Neurotoxins, released from the burn eschar and circulating in the blood stream, may also trigger neuropathies in areas distant from the cutaneous burn [31,32].

## 4. Discussion

Many advances in the treatment of patients with full-thickness burn wounds have been developed in the past decade, leading to a significant increase in quality of life [33]. Although the advancements in surgical reconstructive methods and intensive care management in the treatment of burn patients have been game changing, little attention has been paid to regaining sensibility in the burn areas affected. This is reflected by the paucity of studies on sensory loss and recovery as well as therapeutic approaches. 

In full-thickness skin loss, not only skin and appendages but also nerve endings and various receptors are severed. Conclusively, loss of sensibility in the burned areas was described. In addition, elevation of the sensory threshold in non-burned skin as well as post-burn neuropathies have been reported which have a significant impact on quality of life [9,12,15,16,28,30]. 

Analysis of the current literature revealed that the majority of publications are descriptive in nature. In spontaneously healed superficial burns, the sensibility recovered to normal, except for touch [28]. Most of these reports analyzed adults, while one publication investigated sensitivity in burned children. From these studies, it seems that children might regain sensibility better than adults in terms of different qualities, e.g., mechanoreception and vibration perception. It was hypothesized that additionally grafted pluripotent stem cells and/or mechanoreceptors as part of the grafted skin from other body regions account for better regeneration of touch [34]. Stella et al. identified Merkel cells and free nerve endings in split-thickness skin grafts [26]. 

Post-burn neuropathy and nerve dysfunction distant to the injury site represented another field of investigation in the systemic reaction that follows the burn. Analysis of possible neurotoxic mediators identified elevated TNF alpha, serum nitrite [24,31,32], as well as c-AMP, c-GMP, PGE2, IgF1, and IGFB-3 as possible factors for the so-called nerve insult [35]. Another interesting thesis for altered conduction velocity and misperception at distant sites could be the theory of cortical threshold changes/reorganization [36].

Few reports have addressed the improvement of reinnervation and functional improvement. Contradictory findings on early versus late excision have been published. In animal models, early eschar removal resulted in an improved nerve conduction velocity, indicating regularly functioning nerves [37]. In contrast, Hermanson et al. described that there was no significant difference in sensibility between burns excised and grafted early or late in patients [28]. Reanalysis of studies on early versus late burn eschar excision might give new insight in this topic.

General considerations for improved nerve regeneration in burn patients are ubiquitous measures, such as improving tissue perfusion by various available methods [38], rebalancing metabolism, and preventing small-vessel thrombosis [1,4]. Furthermore, the positioning of the patient to avoid additional stress to peripheral nerves, e.g., by preventing hyperflexion of joints represents pre-condition for less neural strain [37], should not be neglected.

What could be done to minimize nerve damage? 

Future research should focus on optimizing all aspects when treating burn patients with FTBI. Regaining sensibility in most, if not all, sensory quality aspects and, thus, rediscovering their “touch” plays, in the authors’ opinion, a key role in the reintegration of their body image in their everyday life. Possible points of action in the future, representing a valuable adjunct in the treatment of post-burn neuropathy and, most possibly, booster nerve regeneration, could be picked up from the experimental animal studies, which aim to booster nerve regeneration by exogenous-applied substances such as Fujimucin, nerve growth factor, brain-derived nerve factor, recombinant human glial growth factor, nitric oxide and interleukin 1 beta [35,37,39,40,41,42,43], and/or utilizing fibroblast-enriched human skin grafts for more efficiently targeted nerve regrowth.

More studies should focus on supporting sensory reintegration by translating parts of animal studies into human double-blind prospective studies. Cortical reorganization and threshold changings could be antagonized or altered positively by cortical sensory training to prevent or reduce malsensation during the post-burn healing period.

Preventing and antagonizing post-burn mediators into the systemic pathways, which counteract negatively with nerve regeneration by applying intravascular neutralizer, could represent a possible key factor to prevent or reduce peripheral nerve damage, peripheral neuropathies, and neuropathic pain, and promote nerve outgrowth in the burn-wound area in the future.

Most importantly, burn units, physical therapies, and rehabilitative facilities ought to include specific, custom-made central and peripheral sensory training sessions in an interdisciplinary manner.

A limitation of our review is the heterogeneity of the studies. Several animal studies have revealed promising results and are awaiting being adopted in prospective human studies in the future.

## 5. Conclusions

Regaining sensibility in burn patients represents an essential part of a holistic burn-patient treatment protocol. Promising retrospective human and various animal studies reveal that post-burn-injury nerve conduction velocity reduction can be reduced, or even prevented, when excising affected burn areas within a very short time, and sensory nerve regeneration can be boosted by specific mediators. Circulating post-burn mediators ought to be intercepted to reduce general negative systemic effects, including peripheral neuropathy in areas not involved in the injury. In addition, utilizing enriched human skin grafts and exogenous-applied nerve boosters, such as various growth factors, could represent valuable therapeutic adjuncts in a holistic therapeutic concept.

Burn units and physical therapy institutions should expand their burn treatment protocols by including central and peripheral sensory training with various stimuli, e.g., stroking, vibration, and temperature-emitting devices.

## Supplementary Materials

The following supporting information can be downloaded at: https://www.mdpi.com/article/10.3390/ebj3020032/s1, Table S1: Title: Human and Animal Studies Literature Overview in Alphabetical Order.
