# Supplementary material for: “Out of Touch”—Recovering Sensibility after Burn Injury: A Review of the Literature
Source: Eur Burn J. 2022 Jun 14;3(2):370–6. doi: 10.3390/ebj3020032 (PMC11575383; doi:10.3390/ebj3020032)
Supplement: Supplementary file 1 [file ebj-03-00032-s001.zip › ebj-1703561-supplementary.pdf]

Table S1: Human and Animal Studies Literature Overview in Alphabetical Order

Human Studies in alphabetical order	Year	Journal	Study Type
Barret JP, Herndon DN. Modulation of inflammatory and catabolic responses in severely burned children by early burn wound excision in the first 24 hours.	2003	Arch Surg	Retrospective Analysis
Coert JH. Pathophysiology of nerve regeneration and nerve reconstruction in burned patients.	2010	Burns	Pathopghysiology Review
Fuchs S. Klinische Studie : Regenerationspotential der Hautsensibilität nach thermischen Hautschädigungen im Kindesalter Clinical trial : regeneration of skin perception after deep-degree burns in childhood.	2017	Handchir Mikrochir Plast Chir	Retrospective Analysis
Helm PA, Johnson ER, Carlton AM. Peripheral neurological problems in the acute burn patient.	1977	Burns	Retrospective Analysis
Henderson B, Koepke GH, Feller I. Peripheral polyneuropathy among patients with burns.	1971	Archives of Physical Medicine and Rehabilitation	Retrospective Analysis
Hermanson A, Jonsson C, Lindblom U. Sensibility after burn injury.	1986	Clin Physiol	Retrospective Analysis
Khedr EM. Peripheral neuropathy in burn patients.	1997	Burns	Retrospective Analysis
Kowalske K, Holavanahalli R, Helm P. Neuropathy after burn injury.	2001	J Burn Care Rehabil	Retrospective Analysis
Lee MY, Liu G, Kowlowitz V et al. Causative factors affecting peripheral neuropathy in burn patients.	2009	Burns	Retrospective Analysis
Malenfant A, Forget R, Amsel R et al. Tactile, thermal and pain sensibility in burned patients with and without chronic pain and paresthesia problems.	1998	Pain	Retrospective Analysis
Malenfant A, Forget R, Papillon J. et al. Prevalence and characteristics of chronic sensory problems in burn patients.	1996	Pain	Retrospective Analysis
Marquez S, Turley JJ, Peters WJ. Neuropathy in burn patients	1993	Brain	Retrospective Analysis

Nedelec B, Hou Q, Sohbi I et al. Sensory perception and neuroanatomical structures in normal and grafted skin of burn survivors.	2005	Burns	Retrospective Analysis
Sepulchre C, Moati F, Miskulin M et al. Biochemical and pharmacological properties of a neurotoxin protein isolated from the blood serum of heavenly burned patients.	1979	J Pathol	Clinical Study
Stella M, Calcagni M, Teich-Alasia S, Ramieri G, Cellino G, Panzica G. Sensory endings in skin grafts and scars after extensive burns.	1994	Burns	Retrospective Analysis
Strong AL, Agarwal S, Cederna PS, Levi B. Peripheral neuropathy and nerve syndromes in burns.	2017	Clin Plast Surg	Review
Tsolakidis S, Rosenauer R, Schmidhammer R, Pallua N, Rennekampff HO. Wireless microcurrent stimulation improves blood flow in burn wounds.	2021	Burns	Prospective, non-randomized interventional study
Ward RS, Tuckett RP. Quantitative Threshold Changes in Cutaneous Sensation of Patients with Burns.	1991	J Burn Care Rehabil	Retrospective Analysis

**Animal Studies in alphabetical order**	**Year**	**Journal**	**Study Type**
Brauer R.O., Spira M. Full-thickness burns as source for donor graft in the pig.	1950	Plast & Reconst Surg	Experimental
Higashimori H, Carlsen RC, Whetzel TP. Early excision of a full-thickness burn prevents peripheral nerve conduction deficits in mice.	2006	Plast Reconstr Surg	Experimental
Kadanoff D. Histologische Untersuchungen über die Regeneration sensibler Nervenendigungen in Hauttransplantaten.	1925	Klinische Wochenschrift	Anatomical/Histological Study

Kim C, Martyn J., Fuke. Burn injury to trunk of rat causes denervation-like responses in the gastrocnemius muscle.	1988	Br J Pharmacol	Experimental
Morellini NM, Fear MW, Rea S, West AK, Wood FM, Dunlop SA. Reinnervation and nociception after burn injury.	2012	Wound Repair Regen	Experimental
Saffari TM, Schüttenhelm BN, van Neck JW, Holstege JC. Nerve reinnervation and itch behavior in a rat burn wound model.	2018	Wound Repair Regen	Experimental
Tomera JF, Martyn J. Mediators of burn-induced neuromuscular changes in mice.	1989	Br J Pharmacol	Experimental
